# Life Cycle Assessment for Proton Conducting Ceramics Synthesized by the Sol-Gel Process

**DOI:** 10.3390/ma7096677

**Published:** 2014-09-16

**Authors:** Soo-Sun Lee, Tae-Whan Hong

**Affiliations:** Department of Materials Science and Engineering/Research Center of Sustainable Eco-Devices and Materials (ReSEM), Korea National University of Transportation, Daehak-ro 50, Chungju, Chungbuk 380-702, Korea; E-Mail: tjgona@ut.ac.kr

**Keywords:** life cycle assessment, BZY20, BCY10, SCZY, sol-gel

## Abstract

In this report, the environmental aspects of producing proton conducting ceramics are investigated by means of the environmental Life Cycle Assessment (LCA) method. The proton conducting ceramics BaZr_0.8_Y_0.2_O_3-δ_ (BZY), BaCe_0.9_Y_0.1_O_2.95_ (BCY10), and Sr(Ce_0.9_Zr_0.1_)_0.95_Yb_0.05_O_3-δ_ (SCZY) were prepared by the sol-gel process. Their material requirements and environmental emissions were inventoried, and their energy requirements were determined, based on actual production data. This latter point makes the present LCA especially worthy of attention as a preliminary indication of future environmental impact. The analysis was performed according to the recommendations of ISO norms 14040 and obtained using the Gabi 6 software. The performance of the analyzed samples was also compared with each other. The LCA results for these proton conducting ceramics production processes indicated that the marine aquatic ecotoxicity potential (MAETP) made up the largest part, followed by fresh-water aquatic ecotoxicity potential (FAETP) and Human Toxicity Potential (HTP). The largest contribution was from energy consumption during annealing and calcinations steps.

## 1. Introduction

A large number of proton conducting ceramics based on SrCeO_3_, BaCeO_3_ have been developed for many applications [1]. These peroveskite-type oxides are used as solid membranes, in hydrogen sensors and as electrode materials for fuel cells [2], because of their appreciable proton and electronic conductivities [3].

The most widely used synthesissynthetic techniques for these materials have been solid state reaction and sol-gel methods. Solid state processes require high temperature and small particle sizes and have difficulty producing homogeneous composites. In contrast, the sol-gel method can produce high purity materials with good homogeneity at low temperatures [4], but it can be expensive relative to the solid-state reaction approach.

In assessing the sol-gel method for proton conducting ceramics, it is important to consider the technology, economic cost, and environmental impacts. However, to data few studies have made a life cycle assessment of the sol-gel process.

Life Cycle Assessment (LCA) is a technique which can be usefully integrated with technical and, economic assessments of processes. The main aim of LCA is to calculate the environmental impacts of products or processes over their full life-cycle. Such results enable a fair comparison between products or processes and can also contribute to designing products which have a minimal environmental impact. Issues involving toxic emissions or waste can be solved by replacing materials or processes, as can issues impacts related to raw materials and consumed energy.

To synthesize doped perovskites such as BZY20 (BaZr_0.8_Y_0.2_O_3–δ_), BCY10 (BaCe_0.9_Y_0.1_O_2.95_), and SCZY (Sr(Ce_1−x_Zr_x_)_0.95_Yb_0.05_O_3−δ_), rare earth metal are used [5,6,7], including for example, cerium and yttrium . Because the demand for rare earth metals for many application is high in proportion to their small worldwide deposits resource depletion should also be considered when assessing environmental impact.

Thus, this paper aims to present the main results of an LCA applied to the synthesis of proton conducting ceramics (BZY20, BCY10, SCZY) by the sol-gel method, focusing on the main environmental problems of ceramic production, and the opportunities for improvement derived from this analysis. The presented LCA study was performed with the ISO 14040 series.

## 2. Life Cycle Impact Assessment (LCIA)

The LCA is an ISO standardized method (ISO 14040-14044). The quality of an LCA depends on having an accurate description of the production process to be analyzed. It is necessary to collect and interpret that process data to learn where each stage of a life cycle properly begins and ends. In this study, to determine the environmental performance of the processes being studied, a combined Life Cycle Impact Assessment method developed at the University of Leiden, the so called CML (Centrum voor Milieuweten schappen (Institute of Environmental Sciences)) method was used. The CML method defines several impact categories for emissions and for resource consumption as problem-oriented (mid-points) [8]. This method then groups the by-products, emissions and resource consumption caused by the targeted processes into specific environmental impacts, such as global warming potential, acidification potential, ozone depletion potential, *etc*. And these categories are expressed using the reference unit like as kg CO_2_, SO_2_ equivalent. In addition, normalization is applied to compare midpoint impacts.

### 2.1. Goal and Scope

The goal of conducting an environmental impact assessment of the sol-gel process of BZY20, BCY10, and SCZY is to get a clear understanding of which different materials and process steps have the potential to cause most of the environmental burdens. The assessment can also evaluates potential damage to human health. The analysis results will then be used to suggest how to lower the overall environmental impact of the Sol-gel process. This study is a ‘gate-to-gate’ assessment. The system boundaries are the synthesis of the BZY20, BCY10, SCZY powder including the sol-gel process of raw materials and heat treatment, except for the raw material stage and the transport step

**Figure 1:**
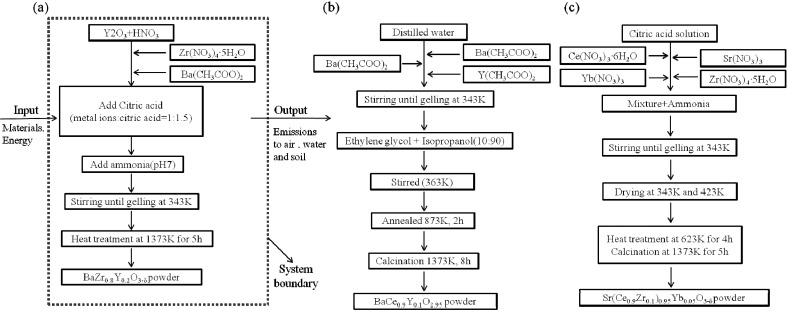
Flow charts of experimental procedures of (**a**) BZY20 (BaZr_0.8_Y_0.2_O_3–δ_); (**b**) BCY10 (BaCe_0.9_Y_0.1_O_2.95_); (**c**) SCZY (Sr(Ce_1−x_Zr_x_)_0.95_Yb_0.05_O_3−δ_).

Figure 1 shows a description of the system and each phase. Data was collected from two stages, sol-gel and heat treatment. The function is defined as a material for the hydrogen membrane, hydrogen gas sensor, hydrogen extractor and fuel cells. Thus, the functional unit of the system was 1 g of powder for each material. All raw materials, wastes, and emissions are based on this functional unit. The electricity considered a power production relies on fossil fuels, price assumed to be from mixed power sources in the Netherlands.

### 2.2. Inventory Analysis

Inventory data for the synthesis of the proton conducting ceramics are presented in Table 1. The inventory data were collected from the literature [5,6,7] and from laboratory experiment, and Ecoinvent Database v2.2 (Swiss Centre for Life Cycle Inventories, Technoparkstrasse, Zürich, Switzerland, 2010) was used [9]. For investigation of the production of proton conducting ceramics, the software Gabi 6 was used. Gabi is a life cycle analysis program and its database contains ecoinvent data for energy, material, and transport systems. Moreover, it can evaluate life cycle environmental, cost, and social profiles of products, processes, and technologies [10].

**Table 1:** Life cycle inventory for the synthesis of BZY20 (BaZr_0.8_Y_0.2_O_3–δ_), BCY10(BaCe_0.9_Y_0.1_O_2.95_) and SCZY(Sr(Ce_1−x_Zr_x_)_0.95_Yb_0.05_O_3−δ_).

Classification	BZY20	BCY10	SCZY
**Raw-materials**	Ammonia (3.6 g)	Barium (0.6 g)	Ammonia (0.6 g)
	Barium (1.3 g)	Cerium (1.0 g)	Cerium (1.9 g)
	Nitric acid (13.7 g)	Yttrium (0.1 g)	Citric acid (2.9 g)
	Yttrium (0.2 g)	Ethylene glycol (0.2 g)	Strontium (1.1 g)
	Zirconium (0.9 g)	Isopropanol (1.5 g)	Ytterbium (1.1 g)
	Distilled water (21.5 g)		Zirconium (1.1 g)
**Electrical equipment**	Running time (h)	Running time (h)	Running time (h)
**Drying oven**	48 (57.6 kWh)	2 (3.8 kWh)	24 (28.8 kWh)
**Electric furnace**	5 (25 kWh)	8 (40 kWh)	9 (945 kWh)
**Emissions to water**	Ammonia (3.6 g)	Ethylene glycol (0.2 g)	Ammonia (0.6 g)
	Nitric acid (13.7 g)		
**Emissions to air**		Isopropanol (1.5 g)	

When an exact dataset could not be used, we chose an appropriate proxy [11,12]. It should be noted that, for the heat treatment and calcination process, the three s samples had different energy requirements. The first stage of sol preparation involved mixing the chemicals together. Then this mixture was stirred until it turned into a gel.

## 3. Results

Classification, characterization and normalization stages were undertaken in this study. Results of the characterization calculated per 1g of synthesized BZY20, BCY10 and SCZY are shown in Table 2.

**Figure 2:**
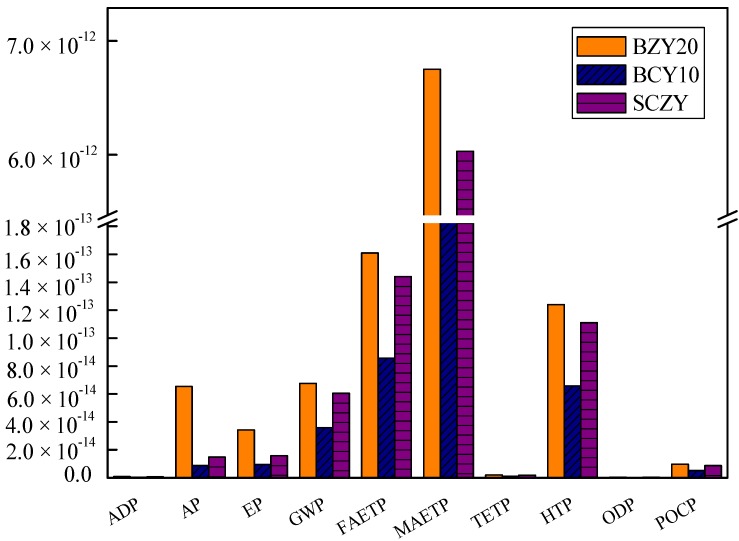
Normalized impacts of BZY20, BZY10 and SCZY the according to CML 2001.

The normalization is based on the national inventory of world impact. The normalized results are displayed in Figure 2. BZY20 has the highest impacts, followed by SZCY, and BCY10. The graphs show that the normalized impact is similar for all three samples in terms of marine aquatic ecotoxicity potential (MAETP) made up the largest part among the tem environmental impact categories followed by fresh-water aquatic ecotoxicity potential (FAETP) and Human Toxicity Potential (HTP). The major environmental impact of these materials is due to the use of electricity (the NL electricity mix). The majority of the energy consumption occurs during the annealing and calcinations steps. Among the three powders, the impacts of BCY10 are lower compared to the others, because the electricity needed is lower than that of the other processes (157.68 MJ). Thus, temperature and holding time are potential areas for reducing the environmental impact.

**Figure 3:**
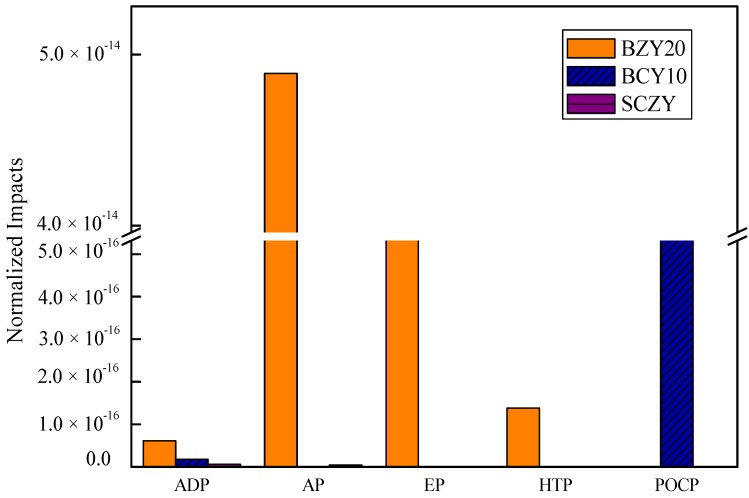
Normalized impacts of BZY20, BZY10 and SCZY (without electricity).

Figure 3 shows ADP (abiotic resource depletion), AP (aciedification potential), EP (eutrophication potential), HTP (Human Toxicity Potential), and POCP (Photochem. Ozone Creation Potential) values for the produced proton conducting ceramics when the impact of electricity is not considered for a material life cycle assessment. In the case of BZY20, the highest impact is AP, followed by EP, HTP and ADP. In Table 3, we show the contributors to AP are nitric acid (51%), which is one of the stronger acidic solutions, and ammonia (49%). Thus, it has negative impacts on the environment and experimenters. Ammonia is used in a neutralization reaction, and affects the EP and HTP impact. The contributors to the ADP impact are barium, zirconium and yttrium rare metals.

In the case of SCZY, the highest impact is ADP, followed by AP, EP and HTP impacts. Like BZY20, SCZY also involves nitric acid and ammonia. However, with BCY10, acidic and basic solutions are not used, which mainly explains its low impact. BCY10 impacts two category indicators (ADP and POCP). The contributors to PCOP are isopropanol and ethylene glycol. The results indicate that the choice of neutralization reaction material has a significant impact.

These results can be useful in process decisions by providing a quantifiable basis for potential improvements in environmental performance of sol-gel processes. In particular, reducing the use of acidic and basic solutions would be favorable improvements for the environment and humans. In addition, considering the industrial scale, one of the advantages of Sol-gel process is relatively uncomplicated to transfer at an industrial scale [13]. Thus, if this method is applied to a large scale, the potential effect is not large. Instead, if the process is improved efficiently, the energy consumption for stage of mass production could be expected to decrease.

**Table 2:** Characterized impacts of the production of BZY20, BCY10 and SCZY.

Impacts category	Unit	Life cycle environmental impacts		
		BZY20	BCY10	SCZY
**Abiotic depletion (ADP)**	kg Sb−Eq	1.58 × 10^−7^	8.10 × 10^−8^	1.31 × 10^−7^
**Acidification potential (AP)**	kg SO_2_−Eq	1.56 × 10^−2^	2.08 × 10^−3^	3.50 × 10^−3^
**Eutrophication potential (EP)**	kg PO_4_^−3^−Eq	5.43 × 10^−3^	1.48 × 10^−3^	2.50 × 10^−3^
**Global warming potential (GWP 100 years)**	kg CO_2_−Eq	2.83 × 10^0^	1.50 × 10^0^	2.53 × 10^0^
**Fresh-water aquatic ecotoxicity potential (FAETP)**	kg DCB−Eq	3.82 × 10^−1^	2.02 × 10^−1^	3.41 × 10^−1^
**Marine aquatic ecotoxicity potential (MAETP)**	kg DCB−Eq	1.31× 10^3^	6.95 × 10^2^	1.17 × 10^3^
**Terrestrial ecotoxicity potential (TETP)**	kg DCB−Eq	2.20 × 10^−3^	1.17 × 10^−3^	1.97 × 10^−3^
**Human toxicity potential (HTP)**	kg DCB−Eq	3.20 × 10^−1^	1.70 × 10^−1^	2.86 × 10^−1^
**Ozone layer depletion potential (ODP)**	kg R11−Eq	1.06 × 10^−7^	5.65 × 10^−8^	9.50 × 10^−8^
**Photochem. ozone creation potential (POCP)**	kg C_2_H_4_−Eq	3.57 × 10^−4^	5.29 × 10^−4^	3.19 × 10^−4^

**Table:** 

Impact category	BZY20	BCY10	SCZY
ADP	6.16 × 10^−17^	1.86 × 10^−17^	6.44 × 10^−18^
AP	4.89 × 10^−14^		4.65 × 10^−18^
EP	1.66 × 10^−14^		1.54 × 10^−18^
HTP	1.39 × 10^−16^		2.7E-20
POCP		9.24 × 10^−15^	

**Table 3:** Contribution to Environmental Impacts.

Impact category	BZY20	BCY10	SCZY
**ADP**	Barium (60%)	Barium (99%)	Strontium (56%)
	Zirconium (39%)		
	Yttrium (1%)	Yttrium (1%)	Zirconium (44%)
**AP**	Nitric acid (51%)	-	Ammonia (100%)
	Ammonia (49%)		
**EP**	Nitric acid (52%)	-	Ammonia (100%)
	Ammonia (47%)		
**HTP**	Ammonia (100%)	-	Ammonia (100%)
**POCP**	-	Isopropanol (82%)	-
		Ethylene glycol (18%)	

## 4. Conclusions

In this paper, the environmental impacts of the synthesis of proton conducting (BZY20, BCY10 and SCZY) materials by the sol-gel method were quantified and described using a life cycle analysis. Several conclusions are drawn from this study.

(1) BZY20 was found to have the highest impacts, followed by SZCY, and BCY10. The normalized results for BZY20 showed marine aquatic ecotoxicity potential (MAETP) made up the largest part followed by fresh-water aquatic ecotoxicity potential (FAETP) and Human Toxicity Potential (HTP). Both BCY10, SCZY showed similar results. The level of energy consumption of the annealing and calcinations steps produced the largest contribution. Thus, temperature and holding time are potentially key targets for reducing the environmental impact.
(2) After excluding the impact of electricity, a material life cycle assessment was conducted. As in the previous results, BZY20 was found to have the highest impacts, followed by SZCY, and BCY10. This is because the use of acidic and basic solutions affected AP (acidification potential) and EP (eutrophication potential). The use of materials such as zirconium and yttrium affected the ADP (abiotic resource depletion) impact. The results indicate that the choice of material (rare metal and solution) has a significant impact. In addition, if the process is improved efficiently, the energy consumption for stage of mass production could be expected to decrease.
